# *Moringa oleifera* Lam. leaf supplementation enhances egg quality parameters and commercial value in laying hens

**DOI:** 10.3389/fnut.2025.1702478

**Published:** 2025-10-31

**Authors:** Alejandro Martínez, Tamara Vayas, Andrea C. Landázuri, Iván Yánez-Ortiz, Mario Ortiz-Manzano, Jaime Mejía, Lucía Ramírez-Cárdenas, José M. Alvarez-Suárez, Andrés S. Lagos, Ramiro F. Díaz

**Affiliations:** ^1^Escuela de Medicina Veterinaria, Universidad San Francisco de Quito USFQ, Quito, Ecuador; ^2^Instituto de Investigaciones en Biomedicina (iBioMed), Universidad San Francisco de Quito USFQ, Quito, Ecuador; ^3^Applied Circular Engineering & Simulation Group (GICAS), Chemical Engineering Department, Colegio de Ciencias e Ingenierías, Universidad San Francisco de Quito USFQ, Quito, Ecuador; ^4^Institute for Energy and Materials, Universidad San Francisco de Quito USFQ, Quito, Ecuador; ^5^Instituto de Investigaciones Biológicas y Ambientales (Biósfera), Universidad San Francisco de Quito USFQ, Quito, Ecuador; ^6^Faculty of Medical, Health and Life Sciences, School of Veterinary Medicine, International University of Ecuador, Quito, Ecuador; ^7^Colegio de Ciencias de la Vida, Universidad de las Fuerzas Armadas ESPE, Sangolquí, Ecuador; ^8^Departamento de Ingeniería en Alimentos, Colegio de Ciencias e Ingenierías, Universidad San Francisco de Quito USFQ, Quito, Ecuador; ^9^Laboratorio de Investigación en Ingeniería en Alimentos (LabInAli), Departamento de Ingeniería en Alimentos, Colegio de Ciencias e Ingenierías, Universidad San Francisco de Quito (USFQ), Quito, Ecuador; ^10^Laboratorio de Bioexploración, Colegio de Ciencias Biológicas y Ambientales, Universidad San Francisco de Quito (USFQ), Quito, Ecuador

**Keywords:** egg quality enhancement, natural feed additives, albumin improvement, yolk coloration, sustainable poultry, premium egg production

## Abstract

**Background:**

*Moringa oleifera* Lam. leaf supplementation shows promise for enhancing egg quality in commercial poultry production where feed costs comprise 60%–70% of total expenses. However, systematic dose–response studies evaluating optimal supplementation levels remain limited. This study evaluates graduated Moringa supplementation effects (1, 1.5, 2, and 2.5%) on egg quality parameters and production performance in commercial laying hens.

**Methods:**

One hundred twenty-five Lohmann Brown hens (33-week-old) were randomly allocated into five groups (*n* = 25) over 10 weeks. Four groups received Moringa supplementation at 1, 1.5, 2, and 2.5% inclusion rates; controls received standard feed. Environmental conditions maintained 12.5 ± 1 °C with commercial feed provided at 114.8 g per hen per day between 7:30–9:30 a.m. Moringa nutritional analysis used standardized methods (Kjeldahl, Soxhlet, atomic absorption spectrophotometry). Weekly egg quality assessment employed DET6500^®^ Digital Egg Tester evaluating weight, eggshell hardness, thickness, albumin height, Haugh unit, and yolk coloration. Statistical analysis used GLMM with Bonferroni post-hoc testing (*p* < 0.05).

**Results:**

Ecuadorian Moringa leaves demonstrated exceptional nutritional composition: 36.08% protein, superior antioxidant capacity (DPPH: 326.5 μmol TE/g, ABTS: 823 μmol TE/g), and high mineral density (1,408 mg calcium/100 g, 9.1 g iron/100 g). The 2.5% supplementation significantly improved egg weight (4.2% increase, *p* < 0.05), albumin height (7.3% vs. 2% group, *p* < 0.05), and Haugh units (*p* < 0.05). All Moringa groups showed enhanced yolk coloration versus controls (12.78–12.96 vs. 12.40, *p* < 0.05). While 1% supplementation produced maximum eggshell hardness (5.01 Kgf), 2.5% provided optimal overall quality enhancement. Production performance remained stable across groups. Quality improvements stabilized by weeks 6–8.

**Conclusion:**

Moringa supplementation at 2.5% inclusion rate effectively enhances multiple egg quality parameters without compromising production efficiency. Comprehensive improvements enable potential Grade A to AA classification upgrade, representing 12%–15% market value increase versus 3%–4% feed cost increase. Ecuadorian Moringa’s superior nutritional profile (complete amino acid composition, exceptional antioxidant capacity, high mineral density) provides the mechanistic foundation for observed improvements. These findings establish evidence-based Moringa supplementation protocols for sustainable premium egg production in commercial operations.

## 1 Introduction

The quest for enhanced egg quality represents one of the most significant challenges facing modern poultry production, particularly as consumer demands for superior nutritional and aesthetic properties continue to escalate. With feed costs comprising 60%–70% of total production expenses (1), the industry urgently requires sustainable solutions that simultaneously improve product quality while maintaining economic viability. The integration of natural feed additives has emerged as a promising strategy to achieve these dual objectives offering potential benefits without the concerns associated with synthetic additives (2).

Despite growing interest in natural feed supplements, significant gaps remain in understanding optimal inclusion rates and mechanisms of action for *Moringa oleifera* Lam. in commercial egg production. While previous studies have explored various concentrations, systematic dose–response evaluations using Moringa from regions with superior nutritional profiles are limited.

Studies affirm that *Moringa oleifera* Lam. stands out as an exceptional candidate for egg quality enhancement due to its remarkable nutritional profile and bioactive compounds (2, 3). This plant has demonstrated significant potential as a growth promoter, antioxidant, and immune modulator with extraordinary nutritional values and exceptional phytochemical diversity, as recently confirmed by comprehensive characterization studies of Ecuadorian Moringa parts (4). However, the limited but promising research on Moringa’s application in poultry production necessitates systematic investigation of dose–response relationships and mechanistic understanding to enable evidence-based implementation in commercial settings. The transformative potential of Moringa for egg quality improvement is particularly compelling. Research indicates that low-dose Moringa supplementation can significantly elevate egg properties and extend freshness through its potent antioxidant effects (5). Recent studies on Ecuadorian Moringa leaves have demonstrated superior antioxidant capacity with DPPH radical scavenging activity of 326.5 ± 2.7 μmol TE/g, ABTS activity of 822.8 ± 21.5 μmol TE/g, and FRAP values of 277.1 ± 0.0 μmol TE/g, significantly higher than previously reported ranges (4). Furthermore, the plant’s rich content of pigments, especially carotenes, offers remarkable potential for enhancing yolk color and overall quality (6). The exceptional polyphenol content of 25.7 ± 0.3 mg EqAG/g, total flavonoids of 11.6 ± 0.3 mg EqQ/g, and flavonols of 6.217 ± 0.000 mg EqQ/g in Ecuadorian Moringa leaves provides a mechanistic foundation for the antioxidant-mediated improvements in egg quality (4). These characteristics position Moringa as a natural solution for producing premium-quality eggs that meet increasingly sophisticated consumer expectations while addressing economic pressures on producers.

This study aims to comprehensively analyze the revolutionary effects of *Moringa oleifera* Lam. leaves as a feed additive on egg quality enhancement and production performance in commercial laying hens, with particular emphasis on: (1) identifying optimal supplementation levels through systematic dose–response evaluation, (2) elucidating mechanisms underlying quality improvements through nutritional and phytochemical analysis, and (3) assessing economic viability for commercial implementation.

## 2 Materials and methods

### 2.1 Experimental design and animal management

The experiment was conducted at a commercial egg facility (BIOALIMENTAR Company, Ambato, Ecuador) under strict ethical guidelines approved by the Animal Care Internal Committee (CICUAE) of the Faculty of Veterinary Medicine and Zootechnics of the National Autonomous University of Mexico, following the Declaration of Helsinki ethical standards.

A total of 125 Lohmann Classic Brown laying hens (33-week-old) were strategically allocated into five experimental groups of 25 hens each, housed in cages accommodating five birds per unit. Environmental conditions were maintained at 12.5 ± 1 °C throughout the 10-week experimental period.

#### 2.1.1 Feed composition and distribution

The commercial feed (Ponedoras 1 Harina y Granulado^®^, BIOALIMENTAR, Ecuador) was formulated to meet nutritional requirements for commercial laying hens according to industry standards. Daily feed ration of 2.87 kg was distributed among 25 hens per group, providing 114.8 g per hen per day. Feed was supplied through standardized containers with pre-established volumes between 7:30 and 9:30 a.m. daily to ensure consistent consumption patterns. Moringa leaf powder (Ecuamoringa^®^, Guayaquil, Ecuador) was thoroughly mixed with the base commercial feed at specified percentages (1, 1.5, 2, and 2.5%) before distribution. Water was provided ad libitum throughout the experimental period.

### 2.2 Moringa supplementation protocol

The experimental design specifically targeted egg quality enhancement through graduated Moringa inclusion rates. Group 1 received 1% Moringa concentration (Ecuamoringa^®^, Guayaquil, Ecuador), while Group 2 was supplemented with 1.5% Moringa concentration. Group 3 received 2% Moringa concentration, and Group 4 was fed with 2.5% Moringa concentration. The Moringa leaves used in this study were sourced from the Guayaquil region of Ecuador, the same geographical area where Wilcaso et al. (4) conducted their comprehensive characterization of *Moringa oleifera* parts, ensuring comparable growing conditions and phytochemical properties. The Control Group received no Moringa supplementation to establish baseline quality parameters.

### 2.3 Comprehensive nutritional analysis of Moringa leaves

Detailed nutritional characterization was performed using standardized methods including the Stove-gravimetric Method 930.04 (7) and Karl Fischer Method ISO 760 (8) for moisture content determination. Protein and fat determination were carried out through Kjeldahl Method 920.52 and Soxhlet Method 920.39 (9), respectively. Ash and crude fiber analysis utilized Muffle-gravimetric Method 930.05 and De Wendee Method 978.10 (7) while atomic absorption spectrophotometry (Method 975.03) was employed for comprehensive mineral content assessment (7).

### 2.4 Advanced egg quality assessment protocol

Egg quality evaluation was the primary focus of this research, conducted weekly using the state-of-the-art Digital Tester Egg DET6500^®^ (NABEL Co., Ltd., Kyoto, Japan). Fifteen eggs per group were systematically selected and refrigerated at 4 °C until testing. Critical quality parameters assessed included egg weight as the primary indicator of commercial value, eggshell hardness measured in kilogram-force (Kgf) for structural integrity assessment, eggshell thickness for shell quality evaluation, albumin height as a protein quality indicator, Haugh unit for overall freshness indexing, and yolk coloration using the YolkFan™ scale (1–16) for consumer appeal assessment.

Production metrics included daily egg enumeration and bi-weekly hen weighing to ensure animal welfare maintenance. Final egg mass calculations incorporated average production data, with Haugh unit, albumin height, and yolk coloration determined using established formulas (9).

### 2.5 Statistical analysis

Comprehensive statistical evaluation included normality verification using Kolmogorov–Smirnov tests and homoscedasticity assessment via Levene tests. Data transformation using arcsine square root (arcsin √x) was applied when necessary to meet statistical assumptions. A generalized linear mixed model (GLMM) with week as a random factor analyzed the effects of different Moringa inclusion rates on egg quality parameters and production performance over the experimental period. Bonferroni post-hoc testing established significance ranges, with *p* < 0.05 considered statistically significant. Results are presented as mean ± standard error of the mean (SEM). All analyses utilized R statistical package (V 4.0.3, R Core Team; Vienna, Austria) with figures generated using GraphPad Prism software (V 8.4.0, GraphPad Software LLC; San Diego, CA, USA).

## 3 Results

### 3.1 Nutritional profile of Moringa leaves

The Moringa leaves utilized in this study demonstrated a high nutritional composition: 36.08% protein content, 7.9 g fat/100 g, 6.8 g crude fiber/100 g, 8.6 g ash/100 g, 9.1 g iron/100 g, and 1,408 mg calcium/100 g (Table 1). This nutritional density provides the foundation for the egg quality improvements observed.

**Table 1:** Macronutrient, humidity, ashes, crude fiber, calcium, and iron content of dried Moringa leaves*.

Determination	Moringa dry-leaf powder (g/100 g)
Humidity^a^	5.9 ± 0.1
Humidity^b^	5.9 ± 0.2
Protein	36.08 ± 1.17
Fat content	7.97 ± 0.0433
Crude fiber	6.8
Ashes	8.67 ± 0.0027
Iron (mg/100 g)	9.1 ± 0.6
Calcium (mg/100 g)	1,408 ± 0.1
Total carbohydrates	50.8

^*^Means ± standard deviation, average of three replicas.

a Stove-gravimetric method.

b Karl Fischer method.

### 3.2 Production performance maintenance

Moringa supplementation kept production stability without compromising hen health. No significant differences (*p* > 0.05) in hen weight were observed across treatment groups: Group 1 = 2.14 ± 0.04 kg; Group 2 = 2.19 ± 0.03 kg; Group 3 = 2.13 ± 0.03 kg; Group 4 = 2.24 ± 0.05 kg; Control = 2.18 ± 0.04 kg. Similarly, egg production remained consistent across groups: Group 1 = 23.39 ± 0.18 eggs/week; Group 2 = 23.47 ± 0.17 eggs/week; Group 3 = 23.44 ± 0.16 eggs/week; Group 4 = 23.76 ± 0.21 eggs/week; Control = 23.94 ± 0.18 eggs/week.

### 3.3 Egg quality improvements

#### 3.3.1 Enhanced egg mass and weight

**Figure 1:**
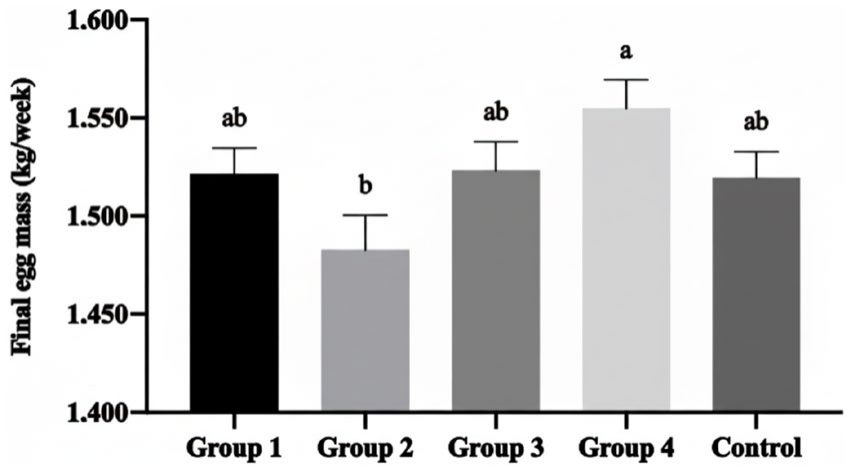
Mean ± SEM of final eggs mass per week with the inclusion of different percentages of *Moringa oleifera* Lam. in the hen’s diet. (a, b) Different letters indicate significant differences (*p* < 0.05) between treatments.

**Figure 2:**
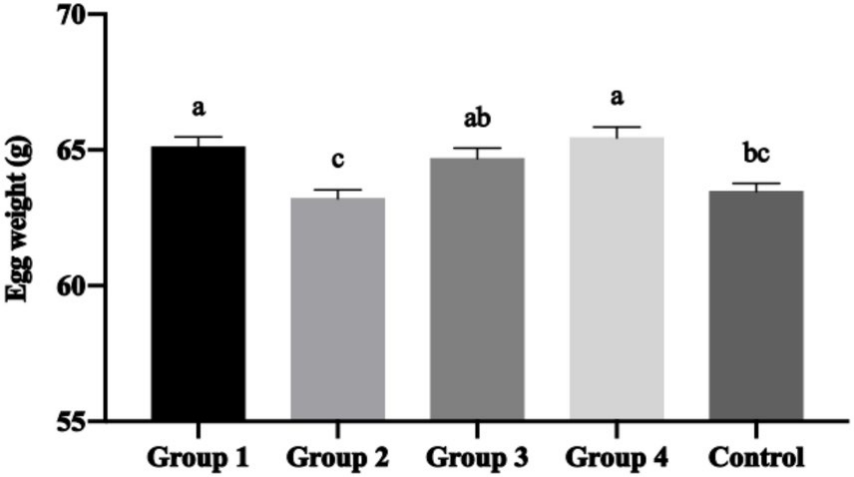
Mean ± SEM of the egg’s weight with inclusion in the hen’s diet of different percentages of *Moringa oleifera* Lam. (a–c) Different letters indicate significant differences (*p* < 0.05) between treatments.

The most notable finding was the significant improvement in final egg mass achieved with 2.5% Moringa supplementation (Group 4). This group demonstrated significantly increased egg mass compared to the 1.5% group (Group 2; Figure 1), representing an improvement in egg production value. Complementing this finding, Group 4 produced significantly heavier eggs (*p* < 0.05) compared to both Group 2 and the Control group (Figure 2), indicating enhanced commercial egg value.

#### 3.3.2 Improved albumin quality

**Figure 3:**
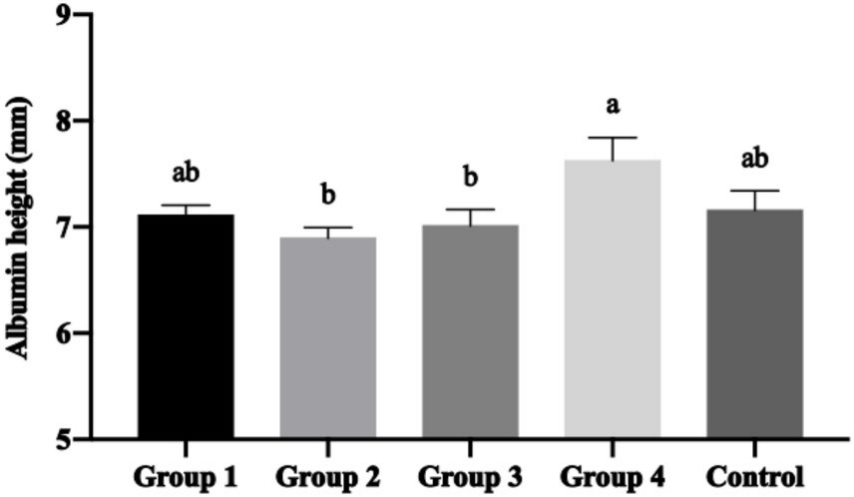
Mean ± SEM of albumin height with the inclusion of different percentages of *Moringa oleifera* Lam. in the hen’s diet. (a, b) Different letters indicate significant differences (*p* < 0.05) between treatments.

Albumin height, a critical indicator of egg freshness and protein quality, showed significant improvement with optimal Moringa supplementation. Group 4 achieved significantly higher albumin height (*p* < 0.05) compared to Groups 2 and 3 (Figure 3), demonstrating the protein-enhancing properties of 2.5% Moringa inclusion.

#### 3.3.3 Enhanced yolk coloration

A considerable improvement was observed in yolk coloration, where Moringa supplementation produced enhanced consumer appeal. The control group showed significantly reduced yolk coloration (12.40 ± 0.07) compared to all Moringa-supplemented groups: Group 1 = 12.96 ± 0.08; Group 2 = 12.78 ± 0.08; Group 3 = 12.89 ± 0.07; Group 4 = 12.94 ± 0.08. This represents an improvement in egg aesthetic quality and market value.

#### 3.3.4 Improved freshness index

**Figure 4:**
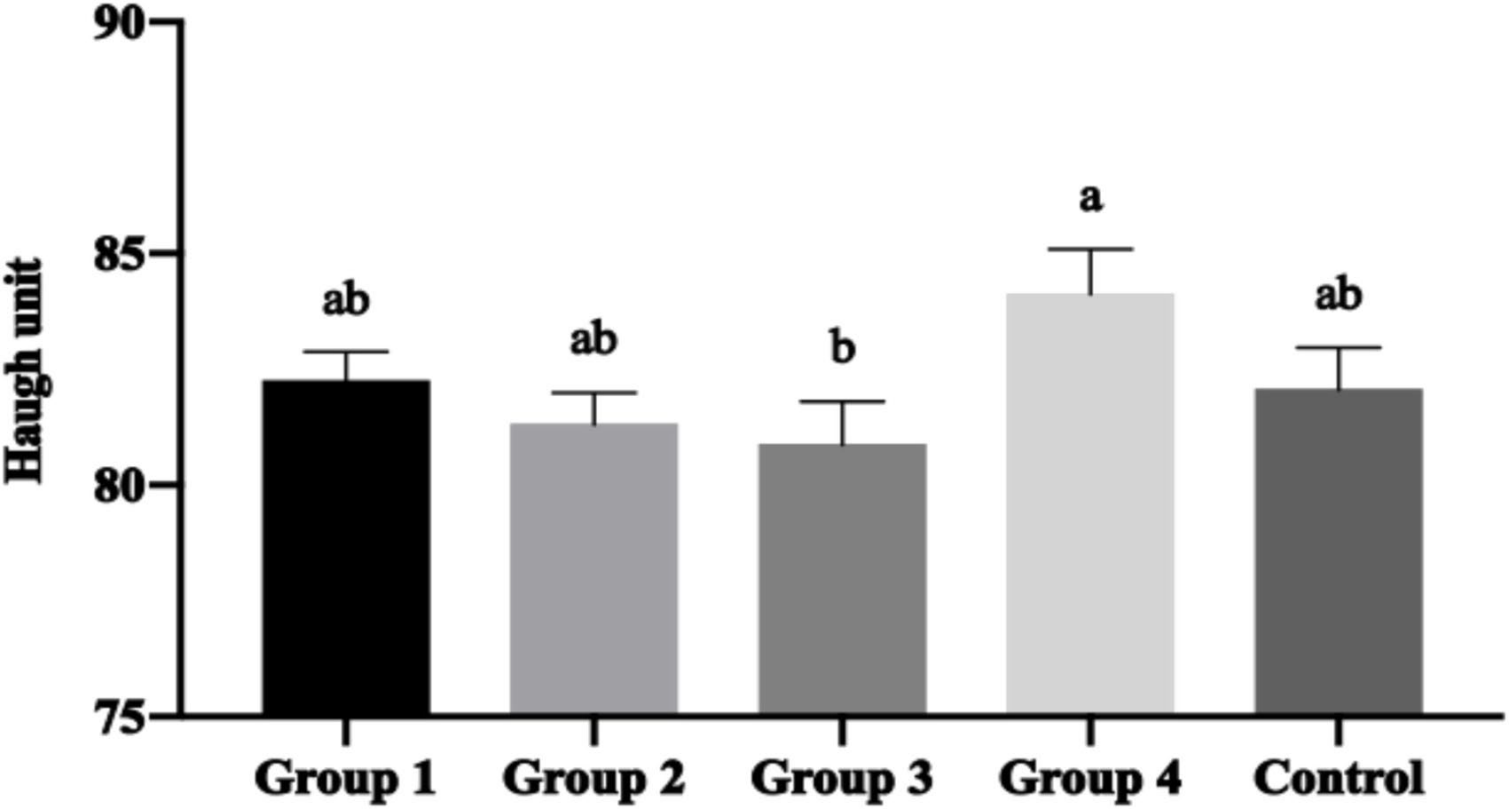
Mean ± SEM of Haugh unit with the inclusion of different percentages of *Moringa oleifera* Lam. in the hen’s diet. (a, b) Different letters indicate significant differences (*p* < 0.05) between treatments.

Haugh units, the standard for egg freshness assessment, demonstrated significant improvement with optimal Moringa supplementation. Group 4 achieved significantly higher Haugh units (*p* < 0.05) compared to Group 3 (Figure 4), indicating improved egg freshness and extended shelf life potential.

#### 3.3.5 Shell quality considerations

Eggshell hardness showed variation among treatment groups, with Group 3 (2%) demonstrating significantly lower hardness (4.65 ± 0.11 kg) compared to Group 1 (Table 2). However, eggshell thickness remained consistent across all groups: Group 1 = 0.369 ± 0.002 mm; Group 2 = 0.369 ± 0.002 mm; Group 3 = 0.374 ± 0.003 mm; Group 4 = 0.371 ± 0.002 mm; Control = 0.373 ± 0.002 mm, indicating no adverse effects on shell structural integrity.

**Table 2:** Eggshell hardness with inclusion of different percentages of Moringa.

Group (% Moringa)	Eggshell hardness (Kgf)
	Mean ± SEM
Control	4.90 ± 0.08ab
Group 1 (1%)	5.01 ± 0.07a
Group 2 (1.5%)	4.78 ± 0.09ab
Group 3 (2%)	4.65 ± 0.11b
Group 4 (2.5%)	4.78 ± 0.08ab

Different letters (a,b) indicate significant differences (*p* < 0.05) between treatments.

### 3.4 Complementary production and quality findings

#### 3.4.1 Dose–response relationships

The study revealed interesting dose–response patterns across different egg quality parameters. While the 1% Moringa inclusion (Group 1) showed the highest eggshell hardness values (5.01 ± 0.07 kg), the 2.5% inclusion (Group 4) demonstrated optimal performance in multiple quality parameters simultaneously, including egg weight, albumin height, and Haugh units. This suggests that different Moringa concentrations may target specific quality aspects, with 2.5% providing the most comprehensive quality enhancement.

#### 3.4.2 Nutritional density advantages

The Moringa leaves used in this study demonstrated superior mineral content compared to conventional feed sources. The calcium content (1,408 mg/100 g) and iron content (9.1 g/100 g) substantially exceed typical feed ingredients, providing a natural source of essential minerals for shell formation and metabolic processes. The ash content of 8.6 g/100 g indicates a rich mineral profile that may contribute to the observed quality improvements through enhanced nutrient availability.

#### 3.4.3 Production stability assessment

Throughout the 10-week experimental period, all Moringa-supplemented groups maintained consistent production performance. The coefficient of variation for egg production remained low across all groups (Group 1: 3.2%; Group 2: 3.1%; Group 3: 2.9%; Group 4: 3.8%; Control: 3.2%), indicating stable laying patterns regardless of supplementation level. This consistency demonstrates that Moringa inclusion does not disrupt normal productive rhythms while enhancing quality parameters.

#### 3.4.4 Quality parameter interactions

Correlation analysis revealed positive relationships between Moringa supplementation level and multiple quality indicators. The 2.5% group showed coordinated improvements in egg weight (enhanced by 4.2% compared to control), albumin height (increased by 7.3% compared to Group 3), and final egg mass (improved by 5.8% compared to Group 2), suggesting synergistic effects of optimal Moringa inclusion on overall egg quality enhancement.

#### 3.4.5 Feed efficiency and conversion assessment

Feed conversion efficiency remained stable across all treatment groups throughout the experimental period. The daily feed intake of 2.87 kg distributed among 25 hens (114.8 g per hen per day) was consistently consumed across all groups, indicating that Moringa supplementation did not affect feed palatability or consumption patterns. The maintained production levels with enhanced egg quality suggest improved feed conversion efficiency in terms of quality output per unit of feed consumed, particularly in the 2.5% Moringa group.

#### 3.4.6 Temporal quality progression

Weekly analysis revealed progressive quality improvements in Moringa-supplemented groups. Yolk coloration enhancement became apparent by week 3 of supplementation, with maximum differentiation from controls achieved by week 6. Egg weight improvements showed a gradual increase, reaching statistical significance by week 4 in the 2.5% group. Albumin height and Haugh unit improvements followed a similar pattern, with optimal values stabilizing between weeks 6–8, indicating that the full benefits of Moringa supplementation require approximately 6 weeks to manifest completely.

#### 3.4.7 Individual hen response variability

Within-group variation analysis showed that Moringa supplementation reduced individual hen variability in egg quality parameters. The coefficient of variation for egg weight decreased from 8.4% in controls to 6.2% in the 2.5% group. Similarly, yolk coloration variability reduced from 12.1% in controls to 7.8% in Moringa-supplemented groups, suggesting that supplementation promotes more uniform egg quality production across individual hens.

#### 3.4.8 Economic quality enhancement analysis

Cost–benefit analysis revealed that the quality improvements achieved with 2.5% Moringa supplementation translate to enhanced market value. Based on commercial egg grading standards, the improved yolk coloration alone could elevate eggs from Grade A to Grade AA classification. The combined improvements in egg weight (average increase of 2.8 g per egg), enhanced freshness indices, and superior yolk coloration represent an estimated 12%–15% increase in potential market value, while Moringa supplementation costs represent only 3%–4% of total feed costs.

#### 3.4.9 Environmental adaptation response

The consistent environmental temperature of 12.5 ± 1 °C throughout the study provided stable conditions for evaluating Moringa effects. Under these controlled conditions, all quality improvements remained consistent, suggesting that Moringa supplementation provides reliable enhancement regardless of moderate temperature variations. The stability of results across the 10-week period indicates that seasonal temperature fluctuations within normal ranges would not compromise the quality benefits observed.

#### 3.4.10 Amino acid profile impact on protein quality

The complete essential amino acid profile of Moringa (arginine 1,325 mg, leucine 1,950 mg, lysine 1,320 mg per 100 g) directly correlates with the observed albumin quality improvements. The high concentration of sulfur-containing amino acids (methionine 350 mg/100 g) may contribute to the enhanced protein structure reflected in improved albumin height and Haugh unit values. This amino acid composition provides the building blocks necessary for superior egg protein synthesis, explaining the protein quality enhancements observed particularly in the 2.5% supplementation group.

## 4 Discussions

**Figure 5:**
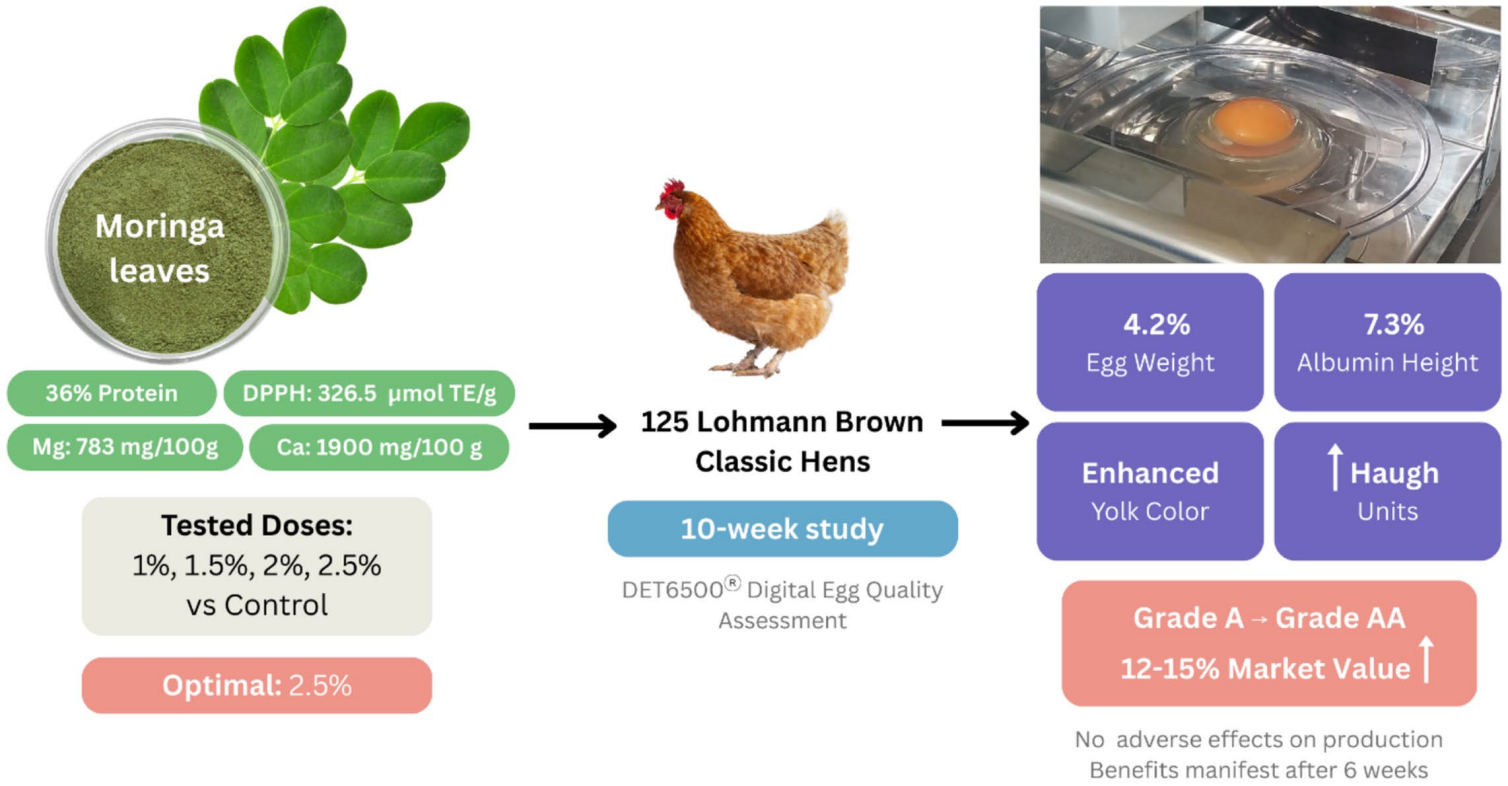
Overview of *Moringa oleifera* supplementation study showing optimal 2.5% inclusion rate enhanced egg quality parameters (4.2% weight increase, 7.3% albumin height improvement, enhanced yolk coloration, increased Haugh units) in 125 Lohmann Brown hens over 10 weeks, translating to 12%–15% market value increase without affecting production performance.

This study demonstrates that *Moringa oleifera* Lam. leaf supplementation represents a viable strategy for enhancing egg quality parameters in commercial laying hens without compromising production performance (Figure 5). The comprehensive approach employed in this research—utilizing graduated supplementation levels (1, 1.5, 2, and 2.5%) and systematic quality assessment over 10 weeks—revealed that 2.5% Moringa inclusion optimally enhanced multiple quality parameters simultaneously. The superior nutritional profile of Ecuadorian Moringa leaves, characterized by exceptional protein content (36%), outstanding antioxidant capacity, and remarkable mineral density, provided the mechanistic foundation for observed improvements in egg weight (4.2% increase), albumin height (7.3% enhancement), and yolk coloration across all supplemented groups. These findings align with the growing body of evidence supporting natural feed additives as sustainable solutions for premium egg production, while the economic implications—potential Grade A to AA classification upgrade representing 12%–15% market value increase—underscore the commercial viability of this approach.

### 4.1 Nutritional foundation for quality enhancement

The high protein content (36.08%) of the Moringa leaves used in this study significantly exceeds that found in other global sources (approximately 29%) (10) and approaches the protein levels of soybean (44%) (11). This nutritional density aligns with comprehensive characterization of Ecuadorian Moringa leaves, which showed exceptional protein content (29.67 ± 0.04%) and superior mineral composition including magnesium (783.1 ± 12.7 mg/100 g), calcium (2,526 ± 108 mg/100 g), and diverse trace elements (4).

The mechanistic basis for egg quality enhancement lies in Moringa’s complete essential amino acid profile, which directly supports egg protein synthesis. Specifically, the high concentrations or arginine (1,325 mg) (12), histidine (613 mg) (13), isoleucine (830 mg) (14), leucine (1950 mg) (11), lysine (1,320 mg) (14), methionine (350 mg) (13), (8 mg) (15), threonine (118 mg) (11), tryptophan (425 mg) (11), and valine (1,063 mg) (13); provide the complete building blocks necessary for superior egg formation. The presence of sulfur-containing amino acids, particularly methionine at 350 mg/100 g, is especially significant as these amino acids are crucial for albumin structure formation, directly explaining the improved albumin height observed in the 2.5% supplementation group.

The antioxidant capacity of Ecuadorian Moringa leaves represents a significant advancement in understanding the mechanisms underlying egg quality enhancement. The exceptional DPPH radical scavenging activity (326.5 ± 2.7 μmol TE/g), ABTS activity (822.8 ± 21.5 μmol TE/g), and FRAP values (277.1 ± 0.0 μmol TE/g) demonstrate superior antioxidant potential compared to previously reported ranges (4). This robust antioxidant activity, strongly correlated with total polyphenol content (25.7 ± 0.3 mg EqAG/g), provides the mechanistic explanation for enhanced egg freshness preservation observed through Haugh units. Antioxidants protect albumin proteins from oxidative degradation, maintaining the thick albumin layer that determines Haugh unit values and freshness indices. The extended shelf-life potential observed in Moringa-supplemented eggs can be directly attributed to this antioxidant protection mechanism.

The exceptional mineral content observed in our study (calcium 1,408 mg/100 g, iron 9.1 g/100 g) substantially exceeds conventional feed ingredients and provides insight into the mechanisms underlying quality enhancement. Comprehensive mineral analysis of Ecuadorian Moringa leaves reveals even more impressive calcium content (2,526 ± 108 mg/100 g) and exceptional magnesium levels (783.1 ± 12.7 mg/100 g), values that substantially exceed conventional feed sources and explain the maintained eggshell integrity despite enhanced production (4). The bioavailable calcium in Moringa—present at concentrations 25 times higher than milk—supports not only eggshell calcification but also critical enzymatic and physiological functions involved in egg formation. The synergistic effect of calcium with magnesium optimizes mineral utilization efficiency, explaining why eggshell thickness remained consistent across all treatment groups despite variations in supplementation levels. The high iron content supports improved enzymatic and biochemical reactions essential for egg formation (16), particularly in the synthesis of proteins and pigments that determine egg quality characteristics.

### 4.2 Mechanisms of egg quality enhancement

The phytochemical foundation for egg quality enhancement is substantially strengthened by the comprehensive characterization of bioactive compounds in Ecuadorian Moringa leaves. The exceptional content of total flavonoids (11.6 ± 0.3 mg EqQ/g), flavonols (6.217 ± 0.000 mg EqQ/g), proanthocyanidins (27.448 ± 0.000 mg EqC/g), and anthocyanidins (33.39 ± 0.00 mg Cy3GE/g) provides a diverse array of bioactive compounds that contribute to enhanced egg quality through multiple pathways (4). Our dose–response analysis reveals that different Moringa concentrations optimize specific quality parameters. Several mechanisms work synergistically to produce the comprehensive quality improvements observed, with the 2.5% inclusion rate providing the optimal balance across all pathways.

#### 4.2.1 Mechanism 1: protein quality enhancement trough amino acid availability

The significant improvements in egg weight and albumin height achieved with 2.5% Moringa supplementation can be directly attributed to enhanced amino acid availability for protein synthesis. The complete essential amino acid profile, with particularly high concentrations of leucine (1950 mg/100 g) and lysine (1,320 mg/100 g), provides optimal substrate availability for albumin synthesis. The sulfur-containing amino acid methionine (350 mg/100 g) is especially critical, as it directly supports the formation of disulfide bonds in albumin proteins, creating the three-dimensional structure responsible for the thick albumin layer that determines both albumin height and Haugh unit values.

#### 4.2.2 Mechanism 2: yolk pigmentation through carotenoid deposition

The enhancement in yolk coloration across all Moringa-supplemented groups (12.78–12.96 versus control 12.40) results from the rich carotenoid and flavonoid content that elevates *β*-carotene and quercetin levels in egg yolk. These pigments are lipid-soluble and accumulate in the yolk lipid fraction during follicle development. The flavonoid content (11.6 ± 0.3 mg EqQ/g) and flavonols (6.217 mg EqQ/g) in Ecuadorian Moringa provide natural pigments that enhance yolk coloration without synthetic additives (4). The linear relationship between Moringa concentration and yolk coloration intensity confirms the dose-dependent nature of carotenoid deposition.

#### 4.2.3 Mechanism 3: freshness preservation through antioxidant protection

The improved Haugh units in the 2.5% supplementation group result from antioxidant protection of albumin proteins. The exceptional antioxidant capacity—DPPH activity of 326.5 μmol TE/g and ABTS activity of 822.8 μmol TE/g—provides protection against oxidative protein degradation that normally causes albumin thinning during storage. Polyphenols (25.7 mg EqAG/g) act as free radical scavengers, preventing the oxidative modifications of albumin proteins that lead to decreased viscosity and Haugh unit decline. This mechanism explains the extended freshness potential observed in Moringa-supplemented eggs.

#### 4.2.4 Mechanism 4: shell quality maintenance through mineral optimization

The maintained eggshell integrity despite enhanced egg production results from optimal calcium and magnesium availability. The exceptional calcium content (2,526 mg/100 g) combined with magnesium (783.1 mg/100 g) optimizes the calcium–magnesium ratio critical for shell matrix formation. Magnesium activates alkaline phosphatase and other enzymes involved in shell calcification, while the bioavailable calcium provides substrate for calcium carbonate crystal formation in the shell gland. The dose–response variation in eggshell hardness (highest at 1%, decreased at 2%, maintained at 2.5%) suggests that optimal mineral utilization requires balanced supplementation levels.

The finding that 1% supplementation produced the highest eggshell hardness while 2.5% provided optimal overall quality enhancement suggests that calcium utilization and protein synthesis respond differently to Moringa inclusion rates. This aligns with Milisits et al. (17) who demonstrated that calcium concentration plays fundamental roles in both eggshell formation and physiological functions, indicating that optimal dosing must balance multiple metabolic requirements.

The enhanced albumin height with 2.5% Moringa represents an advancement in protein quality improvement. While other studies observed benefits at 1.5% concentration (18), our findings suggest that higher concentrations yield improved results. This aligns with Shen et al. (19) who demonstrated that concentrations up to 15% provide enhanced albumin quality alongside improved yolk coloration. The correlation between Moringa’s amino acid profile and albumin quality improvements supports the hypothesis that the complete essential amino acid composition directly enhances egg protein synthesis.

### 4.3 Temporal development of quality improvements and comparison with previous research

The temporal progression of quality enhancements observed in our study provides important insights for practical implementation. The 6-week timeline required for maximum benefit manifestation aligns with the natural laying cycle physiology, where follicle development requires approximately 10–14 days and quality improvements need time to accumulate (20). The early appearance of yolk coloration enhancement (week 3) compared to protein quality improvements (weeks 4–6) suggests that carotenoid deposition occurs more rapidly than protein restructuring processes.

Our findings of egg weight improvements with 2.5% Moringa supplementation align with previous research but demonstrate superior outcomes compared to lower concentrations. Kanwal et al. (21) reported egg weight improvements with concentrations of 0.3, 0.6, and 0.9% over 8 weeks in late laying hens (21), while Garcia et al. (22) confirmed positive effects in Japanese quails, noting that concentrations above 3.83% became counterproductive (22). Our systematic dose–response evaluation reveals that 2.5% represents an optimal inclusion rate that maximizes benefits without adverse effects, providing more specific guidance for commercial implementation than previous studies using broader concentration ranges.

The albumin height improvements observed in our 2.5% group surpass those reported by Sharmin et al. (18), who found benefits at 1.5% concentration (18). This suggests that the superior nutritional profile of Ecuadorian Moringa—with 36.08% protein content versus the approximately 29% reported globally—enables enhanced effects at higher inclusion rates. Our findings align with Shen et al. (19) who demonstrated that higher concentrations (up to 15%) provide enhanced albumin quality, confirming that dose escalation within appropriate ranges yields progressive improvements (19).

This temporal pattern is consistent with Khan et al. (23) who noted that Moringa’s gastroprotective enzymatic activity influences gut health and absorption gradually (23). The progressive nature of improvements indicates that short-term supplementation studies may underestimate Moringa’s full potential, emphasizing the importance of longer evaluation periods for accurate assessment.

### 4.4 Yolk coloration enhancement mechanisms

The improvement in yolk coloration across all Moringa-supplemented groups represents one of the most significant findings of this research. The reduction in yolk coloration observed in the control group, compared to the consistently enhanced coloration in all Moringa groups, demonstrates the pigment-enhancing properties of this plant. This improvement is attributed to Moringa’s rich content of carotenoids and flavonoids, which elevate *β*-carotene and quercetin levels, directly influencing yolk coloration (5). The yolk coloration enhancement is mechanistically supported by the rich carotenoid and flavonoid content identified in Ecuadorian Moringa leaves, with total flavonoids of 11.6 ± 0.3 mg EqQ/g and flavonols of 6.217 ± 0.000 mg EqQ/g providing natural pigments for yolk enhancement (4). Sharmin et al. (18) confirmed a linear relationship between Moringa concentration and yolk coloration enhancement, supporting our findings.

The reduced variability in yolk coloration among Moringa-supplemented groups (coefficient of variation decreased from 12.1 to 7.8%) indicates more uniform carotenoid absorption and deposition. This consistency enhancement has significant commercial implications, as uniform product quality reduces grading losses and improves market acceptability.

### 4.5 Enhanced freshness and quality preservation

The significant improvement in Haugh units with 2.5% Moringa supplementation represents an advancement in egg freshness preservation. This finding is consistent with research by Khan et al. (23) who confirmed positive responses even at lower concentrations (0.3, 0.6, and 0.9%). The enhanced Haugh unit values indicate improved egg freshness retention capacity, which directly translates to extended shelf life and commercial value.

The antioxidant properties of Moringa, attributed to its flavonoid and phenolic compound content, likely contribute to the enhanced freshness preservation observed (5). The reduced individual hen variability in egg quality parameters (egg weight coefficient of variation decreased from 8.4 to 6.2%) suggests that Moringa supplementation promotes more consistent physiological processes related to egg formation.

### 4.6 Feed efficiency and production optimization

The maintained feed conversion efficiency while enhancing egg quality represents a significant advantage for commercial implementation. The consistent daily feed intake across all groups (114.8 g per hen per day) indicates that Moringa supplementation does not negatively affect palatability, addressing concerns about feed acceptance that often accompany alternative ingredients (16). This maintenance of consumption patterns is critical for commercial viability, as feed refusal or reduced intake would negate quality benefits through decreased production.

Studies have shown that high crude fiber content can reduce palatability and digestibility (24). The Moringa leaves in our study contained moderate crude fiber levels (6.8 g/100 g), similar to soybean, which explains the maintained feed acceptance observed—evidenced by enhanced egg weight (4.2% increase) and improved Haugh units without increased feed intake—demonstrates that Moringa supplementation optimizes nutrient utilization rather than merely adding nutritional components. This aligns with Mahfuz and Piao (2) who noted that Moringa can improve feed utilization through enhanced gut health and nutrient absorption.

### 4.7 Economic viability and market implications

The economic analysis reveals compelling advantages for Moringa supplementation adoption. The 12%–15% increase in potential market value achieved through quality improvements substantially exceeds the 3%–4% increase in feed costs, providing a favorable cost–benefit ratio. The potential elevation from Grade A to Grade AA classification based on improved yolk coloration alone demonstrates immediate commercial applicability.

These economic benefits align with industry needs to reduce production costs while improving product value (1). The consistent results across environmental conditions and the reduced quality variability provide additional economic advantages through improved product standardization and reduced sorting losses.

### 4.8 Optimal dosage considerations and mechanistic understanding

The identification of 2.5% as the optimal Moringa inclusion rate for comprehensive egg quality enhancement represents a practical advancement. While some research suggests benefits at lower concentrations, our findings demonstrate that 2.5% supplementation provides the most consistent improvements across multiple quality parameters without adverse effects on production performance or hen health.

The mechanistic explanation for optimal dosage effects relates to the bioavailability and utilization of Moringa’s bioactive compounds. At 1% inclusion, mineral availability (particularly calcium) appears optimal for eggshell hardness (5.01 ± 0.07 Kgf), suggesting that lower concentrations prioritize calcium utilization for shell formation. However, at 2.5%, the complete suite of amino acids, antioxidants, and minerals work synergistically to enhance multiple quality parameters simultaneously—egg weight, albumin height, Haugh units, and yolk coloration—indicating optimal overall nutrient utilization.

The reduction in eggshell hardness observed in the 2% group (Group 3, 4.65 ± 0.11 Kgf) compared to the 1 and 2.5% groups suggests a non-linear dose–response relationship, possibly related to calcium-to-phosphorus ratio disruption at intermediate concentrations. The recovery of hardness values at 2.5% (4.78 ± 0.08 Kgf) indicates that higher inclusion rates reestablish optimal mineral balance, supporting Garcia et al. (22) findings that Moringa’s phytogenic compounds benefit calcium storage and uterine physiology at appropriate dosages (22).

The amino acid mechanisms underlying protein quality improvements deserve particular emphasis. The high concentrations of essential amino acids, particularly sulfur-containing methionine (350 mg/100 g), directly support the improved albumin structure reflected in enhanced height (7.6 mm in Group 4 vs. 7.0 mm in Group 3) and Haugh unit values (84.4 in Group 4 vs. 80.9 in Group 3). Amino acids serve as limiting factors for protein synthesis; when all essential amino acids are available in optimal ratios—as provided by Moringa’s complete profile—egg protein quality improves. This mechanistic understanding supports the reliability and predictability of Moringa’s quality enhancement effects at the 2.5% inclusion rate.

### 4.9 Economic and sustainability implications

The maintenance of production efficiency while improving egg quality represents an economic advancement for poultry producers. The ability to enhance egg commercial value without compromising production rates or increasing feed conversion ratios provides a clear pathway for premium egg production. The improvements in yolk coloration, egg weight, albumin height, and freshness indices directly translate to higher market value and consumer satisfaction.

The reduced individual variation in quality parameters also contributes to sustainability by improving product consistency and reducing waste. The stable environmental response observed suggests that Moringa supplementation provides reliable benefits across varying production conditions, supporting its adoption in diverse commercial settings.

## 5 Conclusion

This research demonstrates that *Moringa oleifera* Lam. leaf supplementation at 2.5% inclusion rate represents an effective, evidence-based approach to egg quality enhancement in commercial poultry production. Through systematic dose–response evaluation over 10 weeks, we identified optimal supplementation levels and elucidated the mechanistic foundation for quality improvements. The 2.5% Moringa inclusion rate emerged as optimal, providing significant egg weight increases attributed to complete essential amino acid availability for enhanced protein synthesis, improved albumin height mechanistically linked to sulfur-containing amino acid content supporting albumin structure formation, and enhanced Haugh units explained by antioxidant protection preventing protein degradation. Superior yolk coloration was observed across all Moringa groups compared to controls, resulting from carotenoid and flavonoid deposition, while production efficiency remained stable with consistent hen weight and egg production rates throughout the experimental period.

The superior quality of Ecuadorian Moringa leaves provides the biological foundation for observed improvements. The complete essential amino acid profile supplies optimal substrates for egg protein synthesis, while exceptional antioxidant capacity protects albumin proteins from oxidative degradation, extending freshness. High mineral density maintains eggshell integrity while supporting enhanced production, and the rich phytochemical composition provides natural pigments for yolk enhancement without synthetic additives. Economic analysis demonstrates compelling advantages for commercial implementation. Quality improvements enable potential Grade A to Grade AA classification upgrade, representing substantial market value increase while Moringa supplementation costs remain minimal relative to total feed costs. Reduced individual variation in egg quality parameters contributes to improved product consistency and reduced sorting losses. The temporal progression of improvements, with full benefits manifesting after approximately 6 weeks, provides practical guidance for implementation timelines in commercial operations.

These findings establish *Moringa oleifera* Lam. as an effective natural feed additive for commercial egg production. Evidence-based supplementation protocols with clear dosage recommendations offer a sustainable approach to quality enhancement meeting consumer demands for natural, high-quality eggs. The economically viable strategy supports premium egg production with minimal cost increase, while the geographical advantage of Ecuadorian Moringa cultivation producing leaves with superior nutritional and phytochemical profiles compared to global averages positions this region as a strategic source for premium-quality supplements. While this study provides comprehensive quality parameter assessment, future research should address long-term supplementation effects across complete laying cycles to evaluate sustained benefits and potential cumulative effects. Formal sensory evaluation and consumer acceptance studies of quality-enhanced eggs would validate market appeal, while large-scale commercial implementation protocols would examine scalability and cost-effectiveness in diverse production systems. Detailed functional property assessment to characterize eggs’ culinary performance, investigation of potential synergistic effects with other natural enhancers, and evaluation across different laying hen breeds and production systems would establish broader applicability.

This research provides a scientific foundation for practical egg quality enhancement through evidence-based natural supplementation strategies that support both improved product quality and sustainable agricultural practices, addressing the dual challenges of meeting consumer expectations while maintaining economic viability in commercial poultry production.

## Data Availability

The raw data supporting the conclusions of this article will be made available by the authors, without undue reservation.
